# Matrix-Free Inexact Preconditioning Techniques for Isogeometric Tensor-Product Discretizations

**DOI:** 10.1007/s10915-026-03253-4

**Published:** 2026-04-27

**Authors:** Michał Ł. Mika, René R. Hiemstra, Dominik Schillinger

**Affiliations:** 1 Institute for Mechanics, Technical University of Darmstadt, Franziska-Braun-Straße 7, Darmstadt, 64287 Germany; 2 Department of Mechanical Engineering, Eindhoven University of Technology, Groene Loper 15, Eindhoven, 5612 AE The Netherlands

**Keywords:** Inexact preconditioning, Kronecker product, Tensor-product splines, Isogeometric analysis, Finite element method

## Abstract

We propose a matrix-free inexact preconditioning strategy for elliptic partial differential equations discretized by the isogeometric Galerkin method on tensor-product spline spaces. We base our preconditioner on an approximation of the discrete linear operator by a sum of Kronecker product matrices. The action of its inverse on a vector of coefficients is approximated by an inner preconditioned conjugate gradient solve. The forward problem is solved by the inexact preconditioned conjugate gradient method. The complexity of the Kronecker matrix-vector products in the inner iteration is lower than the complexity of the matrix-vector products in the forward problem, leading to a reduced number of iterations and significant performance gains. We show the robustness, efficiency and effectiveness of our approach in test problems involving the Poisson equation and linear elasticity, and illustrate the performance gain with respect to preconditioning techniques based on fast diagonalization. The proposed method is implemented in our open-source Julia framework for spline based discretization methods.

## Introduction

In recent decades, the structure of Kronecker product matrices and their properties have been extensively studied and applied in the field of linear algebra, particularly for the solution of linear systems [1]. More recently, Kronecker product techniques gained attention in isogeometric analysis, which primarily uses tensor-product splines. Leveraging the properties of Kronecker product matrices in both standard and isogeometric discretization methods has been shown to accelerate the formation and assembly of linear systems, i.e. by using weighted quadrature rules combined with sum factorization [2–4], fast quadrature rules for immersed methods [5, 6] as well as quadrature rules leveraging interpolation [6–10].

In addition to fast formation and assembly methods, Kronecker product techniques have been applied for the preconditioning of linear systems. The Poisson equation with constant parameters, when discretized using tensor-product spaces on a Cartesian grid, retains a structure that enables efficient numerical treatment. Leveraging this structure, a fast and exact direct solver has been developed to solve the discretized equations efficiently [4]. This solver is further utilized as a preconditioner to accelerate the solution of the Poisson problem on curvilinear grids, where direct methods are computationally expensive [4]. The technique used to develop the fast and exact direct solver is referred to as the fast diagonalization method and is based on diagonalization of generalized Kronecker sums. The fast diagonalization preconditioning strategy extends naturally to the Stokes equations, for which block-diagonal, block-triangular and constrained preconditioners utilizing fast diagonalization have been proposed [11]. Further work on isogeometric Kronecker product preconditioners includes an extension of fast diagonalization to non-symmetric systems, such as isogeometric discretizations with weighted quadrature rules [12], as well as preconditioning of isogeometric mass-matrices [13] and combining weighted quadrature, fast diagonalization and matrix-free matrix-vector products for the solution of the non-linear transient heat equation [14].

A related idea leveraging the tensor-product structure of isogeometric discretizations are low-rank representations of the system matrices and low-rank solvers based on these representations. Mantzaflaris et al. [15, 16] obtain low-rank Kronecker representations of Galerkin matrices and employ these for $L^2$-projection and a Laplace problem using the conjugate gradient solver. High efficiency is attained compared to standard formulation and assembly strategies, particularly when the rank remains sufficiently low. Obtaining a low-rank representation was discussed by Georgieva et al. [17]. Similar ideas were followed by Bünger et al. [18] to solve isogeometric constrained optimization problems and by Montardini et al. [19] for problems in linear elasticity.

The objective of our work is to leverage low-rank Kronecker product representations to improve robustness and efficiency of matrix-free full-rank solvers on tensor-product grids. The construction of our low-rank preconditioners is similar to the construction of low-rank tensor methods in Galerkin-based isogeometric analysis [16]. In their work the authors project the kernel function in the integrand of stiffness and mass matrices onto a spline space and perform low-rank tensor decomposition of the spline coefficients. With that procedure they obtain a separable representation of the kernel that allows for an approximation of the full-rank matrix by a sum of Kronecker product matrices. By controlling the error in the spline projection and the rank of the tensor decomposition they arrive at fast and accurate low-rank solvers. In contrast, we use low-rank Kronecker product representations as a preconditioner to full-rank solvers on tensor-product grids. Furthermore, instead of projecting the kernel, we opererate directly on the data at quadrature points. Since the inverse of a sum of Kronecker product matrice is in general not readily available, we choose to approximate the action of the inverse by an iterative inner linear solver. This choice has implications on the selection of a suitable outer linear solver for the forward problem. In order to accommodate an inexact preconditioning strategy – often referred to as flexible or variable preconditioning – we turn our attention to a class of linear solvers designed with such preconditioners in mind. Notable examples are the inexact preconditioned conjugate gradient method [20–23] (IPCG), the flexible generalized minimum residual method [24] (FGMRES) and the nested GMRES [25] (GMRESR) solver. Further work has been done on flexible linear solvers in recent years [26–29].

In Section 2, we review the properties of Kronecker product matrices, which are essential for the design of our inexact preconditioning strategy. We proceed with a discussion of low-rank approximations to finite element matrices in Section 3. Here, we show the approximation of a stiffness matrix of the Poisson equation. The solvers used in this work are introduced in Section 4. In Section 5, we show the performance of our approach for the Poisson equation and linear elastic problems. A summary and conclusions are given in Section 6.

## Kronecker Product Matrices and Their Properties

Let $\textsf{A} \in \mathbb {R}^{m \times n}$ and $\textsf{B} \in \mathbb {R}^{p \times q}$ denote real valued matrices. The Kronecker product $\textsf{A} \otimes \textsf{B} \in \mathbb {R}^{m \cdot p \times n \cdot q}$ is the matrix

1 $$\begin{aligned} \textsf{A} \otimes \textsf{B} := \begin{bmatrix} A_{11} \textsf{B} &  \ldots &  A_{1n} \textsf{B} \\ \vdots &  &  \vdots \\ A_{m1} \textsf{B} &  \ldots &  A_{mn} \textsf{B} \end{bmatrix}. \end{aligned}$$

Given $\textsf{A}$, $\textsf{B}$, $\textsf{C}$ and $\textsf{D}$ of compatible sizes, the Kronecker product satisfies the properties:

2a $$\begin{aligned} \left( \textsf{A} \otimes \textsf{B} \right) \otimes \textsf{C}&= \textsf{A} \otimes \left( \textsf{B} \otimes \textsf{C} \right)  &   (\text {associativity}) \end{aligned}$$

2b $$\begin{aligned} \left( \textsf{A} \otimes \textsf{B} \right) \left( \textsf{C} \otimes \textsf{D} \right)&= \left( \textsf{A} \textsf{C} \right) \otimes \left( \textsf{B} \textsf{D} \right)  &   (\text {mixed product property}) \end{aligned}$$

2c $$\begin{aligned} \left( \textsf{A} \otimes \textsf{B} \right) ^{-1}&= \textsf{A}^{-1} \otimes \textsf{B}^{-1}  &   (\text {inverse of a Kronecker product}) \end{aligned}$$

2d $$\begin{aligned} \left( \textsf{A} \otimes \textsf{B} \right) ^\mathrm T&= \textsf{A}^\mathrm T\otimes \textsf{B}^\mathrm T  &   (\text {transpose of a Kronecker product}) . \end{aligned}$$

The Kronecker matrices in this work are typically a product of small univariate matrices which are stored in dense format. To highlight that, we denote them by capital sans-serif letters, $\textsf{A}$. In contrast, large matrices not suitable for dense storage are denoted by bold capital letters, $\textbf{M}$. Additionally, we denote vectors by small bold letters, $\textbf{x}$, and multidimensional arrays and tensors by calligraphic letters, $\mathcal {X}$. Indexing into matrices, vectors, arrays and tensors is denoted by square brackets, $[\bullet ]_{ijk}$.

### Kronecker Matrix-Vector Multiplication

Iterative linear solvers typically require only the action of a preconditioner on a vector to be available and implemented. Our preconditioners are defined as a sum of Kronecker matrices, which enables efficient matrix-vector multiplication.

Let $\mathsf {\mathcal {X}} \in \mathbb {R}^{n_1 \times \cdots \times n_d}$ and $\mathcal {Y} \in \mathbb {R}^{m_1 \times \cdots \times m_d}$ be two *d*-dimensional arrays. The vectorization of $\mathcal {X}$ is a linear operation that maps $\mathcal {X}$ to a vector $\textbf{x} \in \mathbb {R}^{n_1\cdots n_d}$ defined by

3 $$\begin{aligned} {[}\textbf{x}]_i := [\mathcal {X}]_{i_1 \ldots i_d}, \text { where } i=i_1 + (i_2-1)n_1 + \cdots + (i_d-1) n_1 \cdot \ldots \cdot n_{d-1}, \end{aligned}$$

with indices $i_k = 1,\ldots ,n_k$, $k=1,\ldots ,d$. If $\mathcal {X}$ is stored in column-major order, the vectorization preserves the storage order, making the transformation a zero-cost operation. Analogously, the vectorized representation of $\mathcal {Y}$ is denoted by $\textbf{y}\in \mathbb {R}^{m_1\cdots m_d}$.

Let $\textsf{D}_k \in \mathbb {R}^{m_k \times n_k}$, $k=1, \ldots , d$, denote a set of *d* univariate matrices. Furthermore, define $N:=n_1\cdots n_d$ and $M:=m_1\cdots m_d$ as the lengths of the vectorized representations $\textbf{x}\in \mathbb {R}^{N}$ and $\textbf{y} \in \mathbb {R}^{M}$, respectively. The algorithmic complexity of the dense matrix-vector product

4 $$\begin{aligned} \textbf{y}&= \left( \textsf{D}_d \otimes \cdots \otimes \textsf{D}_1 \right) \textbf{x} \end{aligned}$$

is $\mathcal O({MN})$ floating-point operations (flops). However, this complexity can be generally substantially reduced by rewriting the product as a sequence of modewise contractions,

5 $$\begin{aligned} {[}\mathcal {Y}]_{i_1 \ldots i_d}&= \sum _{j_d=1}^{n_d} [\textsf{D}_d]_{i_d j_d} \Bigg ( \ldots \Bigg ( \overbrace{ \sum _{j_{2}=1}^{n_2} [\textsf{D}_{2}]_{i_{2}j_{2}} \bigg ( \underbrace{ \sum _{j_1=1}^{n_1} [\textsf{D}_1]_{i_1 j_1} [\mathcal {X}]_{j_1 \ldots j_d} }_{\mathcal {X}^\prime \, \in \; \mathbb {R}^{m_1 \times n_2 \times \cdots \times n_d}} \bigg ) }^{\mathcal {X}^{\prime \prime }\, \in \; \mathbb {R}^{m_1\times m_2 \times n_3 \times \cdots \times n_d}} \Bigg ) \Bigg ). \end{aligned}$$

The complexity then depends on the dimensions of the individual univariate matrices as well as the order in which the modewise contractions are performed. For simplicity, we assume the evaluation order indicated by the brackets in Equation (5). The evaluation of the innermost bracket requires $n_2 \cdots n_d$ matrix-vector multiplications between $\textsf{D}_1$ and a slice of $\mathcal {X}$, namely, one vector slice for each combination of the free indices $j_2,\ldots ,j_d$. A dense matrix-vector product with $\textsf{D}_k$ requires $\mathcal O({m_k n_k})$ flops. Thus, this contraction results in $\mathcal O({m_1 n_1 \cdot n_2\cdots n_d })$ flops and, as indicated by the underbrace, generates a new *d*-dimensional array $\mathcal {X}^{\prime }$ of size $m_1\times n_2 \times \cdots \times n_d$. The evaluation of the second innermost bracket requires $m_1 n_3\cdots n_d$ dense matrix-vector multiplications between $\textsf{D}_2$ and a slice of the array created in the previous stage, namely, one vector slice of $\mathcal {X}^{\prime }$ for each combination of the free indices $i_1,j_3,\ldots ,j_d$. Analogously, this contraction requires $\mathcal O({m_2 n_2 \cdot m_1 n_3\cdots n_d })$ flops and results in a new *d*-dimensional array $\mathcal {X}^{\prime \prime }$ of size $m_1 \times m_2 \times n_3 \times \cdots \times n_d$. This procedure is repeated until all indices $j_k$ are eliminated through contraction with the corresponding univariate matrix $\textsf{D}_k$, $k=1,\ldots ,d$. For this particular contraction order the total flops are

6 $$\begin{aligned} \mathcal {O} \left( \sum _{k=1}^d m_k n_k \prod _{l<k} m_l \prod _{l>k} n_l\right) . \end{aligned}$$

If all $\textsf{D}_k$ are of equal size, $m:= m_1 = \cdots = m_d$ and $n:=n_1 = \cdots = n_d$, and the dimension $d \ll \max (m,n)$, the estimate simplifies to $\mathcal {O} \left( m^d n\right)$ in the case $m > n$, and $\mathcal {O} \left( m n^d\right)$ for $m < n$. Moreover, if $m = n$, the Kronecker product matrix is square and of size $N\times N$, where $N:=n^d$. In that case the complexity of the Kronecker matrix-vector product is $\mathcal O({N^{(d+1)/d}})$ showing that the modewise contraction approach scales nearly linearly with the matrix size, and thus significantly outperforms standard dense matrix-vector multiplication, which scales $\mathcal O({N^2})$ with respect to the matrix size.

In practice, as well as in our implementation, highly optimized linear tensor algebra libraries can be used to evaluate the sequence of contraction such as the Julia package TensorOperations [30], which, among others, take into account the optimal evaluation order [31]. Lastly, we note that the dense storage cost of the univariate matrices $\textsf{D}_k$ is negligible in practical applications.

### Kronecker Sums

Consider a set of *d* square matrices $\textsf{A}_k \in \mathbb {R}^{n_k \times n_k}$, and identity matrices $\textsf{I}_k \in \mathbb {R}^{n_k \times n_k}$, $k=1, \ldots , d$. The Kronecker sum $\textsf{A}_1 \oplus \cdots \oplus \textsf{A}_d \in \mathbb {R}^{(n_1\cdots n_d) \times (n_1\cdots n_d)}$ is the matrix

7 $$\begin{aligned} \begin{aligned} \textsf{A}_1 \oplus \cdots \oplus \textsf{A}_d&:= \textsf{I}_d \otimes \cdots \otimes \textsf{I}_2 \otimes \textsf{A}_1 \\&\quad + \textsf{I}_d \otimes \cdots \otimes \textsf{I}_{3} \otimes \textsf{A}_2 \otimes \textsf{I}_1 \\&\quad + \cdots \\&\quad + \textsf{I}_d \otimes \textsf{A}_{d-1} \otimes \textsf{I}_{d-2} \otimes \cdots \otimes \textsf{I}_1 \\&\quad + \textsf{A}_d \otimes \textsf{I}_{d-1} \otimes \cdots \otimes \textsf{I}_1. \end{aligned} \end{aligned}$$

The Kronecker sum is commutative up to permutation similarity. However, it is neither associative nor distributive with respect to the Kronecker product.

Let $\textsf{M}_k, \; k=1, \ldots , d$, denote a set of *d* symmetric positive definite matrices, e.g. finite element mass matrices. We define the following generalized Kronecker sum,

8 $$\begin{aligned} \begin{aligned} \textsf{A}_1 \mathop {\hat{\oplus }}\cdots \mathop {\hat{\oplus }}\textsf{A}_d&:= \textsf{M}_d \otimes \cdots \otimes \textsf{M}_2 \otimes \textsf{A}_1 \\&\quad + \textsf{M}_d \otimes \cdots \otimes \textsf{M}_{3} \otimes \textsf{A}_2 \otimes \textsf{M}_1 \\&\quad + \cdots \\&\quad + \textsf{M}_d \otimes \textsf{A}_{d-1} \otimes \textsf{M}_{d-2} \otimes \cdots \otimes \textsf{M}_1 \\&\quad + \textsf{A}_d \otimes \textsf{M}_{d-1} \otimes \cdots \otimes \textsf{M}_1. \end{aligned} \end{aligned}$$

A generalized Kronecker sum can always be transformed into a regular Kronecker sum. Consider the Cholesky factorization of the matrix $\textbf{M} = \textsf{M}_d \otimes \cdots \otimes \textsf{M}_1$,

9 $$\begin{aligned} \textbf{M} = \textbf{L} \textbf{L}^\mathrm T= \left( \textsf{L}_d \otimes \cdots \otimes \textsf{L}_1 \right) \left( \textsf{L}^\mathrm T_d \otimes \cdots \otimes \textsf{L}^\mathrm T_1 \right) . \end{aligned}$$

Using the mixed product property of Kronecker product matrices (2b), we may write

10 $$\begin{aligned} \textbf{L}^{-1} \left( \textsf{A}_1 \mathop {\hat{\oplus }}\cdots \mathop {\hat{\oplus }}\textsf{A}_d \right) \textbf{L}^{-\mathrm T} = \hat{\textsf{A}}_1 \oplus \cdots \oplus \hat{\textsf{A}}_d, \end{aligned}$$

where $\hat{\textsf{A}}_k = \textsf{L}_k^{-1} \textsf{A}_k \textsf{L}_k^{-\mathrm T}$, $k=1, \ldots , d$. We emphasize, that not all sums of Kronecker product matrices are a Kronecker sum as defined in (7) and (8).

### Fast Diagonalization of Kronecker Sums

Let $\textsf{A}_k = \textsf{U}_{k} \mathsf {\Lambda }_k \textsf{U}^\mathrm T_{k}$ denote the eigenvalue decomposition of real symmetric matrices $\textsf{A}_k, \; k=1,\ldots , d$. Using $\textsf{I}_k = \textsf{U}^\mathrm T_{k} \textsf{U}_{k}$ and repeated application of the mixed-product property of Kronecker product matrices (2b), it is easy to show that a Kronecker sum has the following eigenvalue decomposition,

11 $$\begin{aligned} \textsf{A}_1 \oplus \ldots \oplus \textsf{A}_d = \big (\textsf{U}_d \otimes \ldots \otimes \textsf{U}_1 \big ) \big ( \mathsf {\Lambda }_1 \oplus \ldots \oplus \mathsf {\Lambda }_d \big ) \left( \textsf{U}^\mathrm T_d \otimes \ldots \otimes \textsf{U}^\mathrm T_1 \right) . \end{aligned}$$

Similarly, the generalized eigenvalue decomposition of a generalized Kronecker sum is found using Equation (10) by multiplying with the Cholesky factor on each side,

12 $$\begin{aligned} \textsf{A}_1 \mathop {\hat{\oplus }}\ldots \mathop {\hat{\oplus }}\textsf{A}_d = \left( \textsf{L}_d \otimes \ldots \otimes \textsf{L}_1 \right) \left( \hat{\textsf{A}}_1 \oplus \ldots \oplus \hat{\textsf{A}}_d\right) \left( \textsf{L}^\mathrm T_d \otimes \ldots \otimes \textsf{L}^\mathrm T_1 \right) . \end{aligned}$$

Recall that $\hat{\textsf{A}}_k = \textsf{L}_k^{-1} \textsf{A}_k \textsf{L}_k^{-\mathrm T}$ and let $\hat{\textsf{A}}_k = \textsf{U}_k \mathsf { \mathsf {\Lambda }}_k \textsf{U}_k^\mathrm T$ denote the eigenvalue decomposition of $\hat{\textsf{A}}_k$. Equation (11) and repeated application of the mixed-product property (2b) together with the definition $\hat{\textsf{U}}_k:= \textsf{L}_k \textsf{U}_k$ gives the generalized eigenvalue decomposition

13 $$\begin{aligned} \textsf{A}_1 \mathop {\hat{\oplus }}\ldots \mathop {\hat{\oplus }}\textsf{A}_d = \left( \hat{\textsf{U}}_d \otimes \ldots \otimes \hat{\textsf{U}}_1 \right) \left( \mathsf {\Lambda }_1 \oplus \ldots \oplus \mathsf {\Lambda }_d \right) \left( \hat{\textsf{U}}^\mathrm T_d \otimes \ldots \otimes \hat{\textsf{U}}^\mathrm T_1 \right) . \end{aligned}$$

It is easy to show that $\textsf{I}_k = \hat{\textsf{U}}_k^\mathrm T\textsf{M}_k^{-1} \hat{\textsf{U}}_k$.

Note, that the decompositions involve Kronecker product and diagonal matrices. Therefore, the inverse matrix can be constructed efficiently by leveraging the Kronecker product inverse property (2c). This technique has been adopted in the fast diagonalization preconditioners [4, 11] and is also a part of our approach.

## Low-Rank Approximations of Finite Element Matrices

Our preconditioning strategy is based on low-rank approximations of discrete operators in finite elements. We start by discussing the Kronecker product structure of the discrete operator for a simple scalar problem with constant data on a unit cube discretized with tensor-product spaces. The model problem is then extended by spatially variable data and a geometry mapping. In this case, the Kronecker product structure of the discrete operator is lost, which requires us to introduce an approximation. In particular, we approximate the geometry and model data at the quadrature points. We describe an automated procedure to construct low-rank approximations as implemented in our finite element framework.

### Variational Formulation of the Poisson Equation

Let us consider the Poisson equation on a bounded domain $\Omega \subset \mathbb {R}^d$ with homogeneous boundary conditions on $\partial \Omega$,

14 $$\begin{aligned} \begin{aligned} -\nabla \cdot (\kappa (\boldsymbol{x}) \nabla u(\boldsymbol{x}))&= f(\boldsymbol{x}) \qquad  &   \text {in} \quad  &   \Omega \\ u&= 0 \qquad  &   \text {on} \quad  &   \partial \Omega . \end{aligned} \end{aligned}$$

Here, $u \in \mathcal {S}$ is a scalar field, the conductivity $\kappa$ is a positive definite matrix in $\mathbb {R}^{d\times d}$, *d* is the model problem dimension and $f \in \mathbb {R}$ is the source term. The variational formulation of (14) is given as

15 $$\begin{aligned} \int _\Omega \kappa \nabla u \cdot \nabla v \,\mathrm dx = \int _\Omega fv\,\mathrm dx \quad \forall v \in \mathcal {V}. \end{aligned}$$

Let $\hat{B}_{\textsf{i}}(\hat{\boldsymbol{x}}) = \Pi _{k = 1}^d \hat{B}_{i_k}(\hat{x}_k)$, $i_k = 1,\ldots ,n_{k}$, be a set of tensor-product basis functions, throughout this work B-splines^1^, on $\hat{\Omega }= [0,1]^d$ indexed by a multi-index $\textsf{i}:= (i_1,\ldots , i_d)$. With the parametrization $\Omega = \boldsymbol{F}({\hat{\Omega }})$ and $\textbf{J} = \textrm{D}\boldsymbol{F}$, we define basis functions on $\Omega$ as $B_{\textsf{i}}(\boldsymbol{x}) = \hat{B}_{\textsf{i}} \circ \boldsymbol{F}^{-1}(\boldsymbol{x})$. We set the trial and test space to $\{B_{\textsf{i}}:\; \textsf{i} = (i_1,\ldots , i_d), 2\le i_k\le (n_k - 1), 1\le k \le d\}$ and thus satisfy the homogeneous boundary conditions. We are particularly interested in the stiffness matrix from the discretized variational formulation which reads

16 $$\begin{aligned} {[}\textbf{K}]_{\textsf{i} \textsf{j}} = \sum _{\alpha =1}^{d} \sum _{\beta =1}^d \int _{\hat{\Omega }} \frac{\partial \hat{B}_{\textsf{i}}}{\partial \hat{x}_\alpha } [\textbf{C}]_{\alpha \beta } \frac{\partial \hat{B}_{\textsf{j}}}{\partial \hat{x}_\beta } \,\textrm{d}\hat{\boldsymbol{x}} \; , \quad \text {where}\quad \textbf{C}(\hat{\boldsymbol{x}}) = \textbf{J}^{-\mathrm T} (\kappa \circ \boldsymbol{F}) \textbf{J}^{-1} \textrm{det}(\textbf{J}). \end{aligned}$$

### Direct Solver

Let us assume that $\boldsymbol{F}$ is the identity map and the conductivity tensor is constant and diagonal. In that case, the data matrix $\textbf{C}$ reduces to a diagonal matrix and the sum in (16) simplifies to

17 $$\begin{aligned} \begin{aligned} {[}\textbf{K}]_{\textsf{i} \textsf{j}}&= \sum _{\alpha =1}^{d} \int _{\hat{\Omega }} \frac{\partial \hat{B}_{\textsf{i}}}{\partial \hat{x}_\alpha } [\kappa ]_{\alpha \alpha } \frac{\partial \hat{B}_{\textsf{j}}}{\partial \hat{x}_\alpha } \,\textrm{d}\hat{\boldsymbol{x}}\\&= \sum _{\alpha =1}^{d} \int _{\hat{\Omega }} \frac{\partial (\hat{B}_{i_1}(\hat{x}_1)\cdots \hat{B}_{i_d}(\hat{x}_d)) }{\partial \hat{x}_\alpha } [\kappa ]_{\alpha \alpha } \frac{\partial (\hat{B}_{j_1}(\hat{x}_1)\cdots \hat{B}_{j_d}(\hat{x}_d)) }{\partial \hat{x}_\alpha } \,\textrm{d}\hat{\boldsymbol{x}} \end{aligned} \end{aligned}$$

By differentiating the products and separating the integrals we obtain

18 $$\begin{aligned} \textbf{K} = \sum _{\alpha =1}^{d} \textsf{A}_{d}^{\alpha } \otimes \cdots \otimes \textsf{A}_{1}^{\alpha } \end{aligned}$$

The *d* univariate matrices $\textsf{A}_{l}^{\alpha }$ of size $n_l \times n_l$, $l = 1,\ldots ,d$, are small stiffness matrices $\textsf{K}_l:= \textsf{A}_{l}^{\alpha }$ if $l=\alpha$,

19 $$\begin{aligned} {[}\textsf{A}_{l}^{\alpha }]_{i_l j_l} := \int _0^1 \frac{\partial \hat{B}_{i_l}(\hat{x}_l) }{\partial \hat{x}_\alpha } [\kappa ]_{\alpha \alpha }^{1/d} \frac{\partial \hat{B}_{j_l}(\hat{x}_l) }{\partial \hat{x}_\alpha } \,\textrm{d}\hat{x}_l \qquad (l = \alpha ) \end{aligned}$$

and small mass matrices $\textsf{M}_l:= \textsf{A}_{l}^{\alpha }$ otherwise,

20 $$\begin{aligned} {[}\textsf{A}_{l}^{\alpha }]_{i_l j_l} := \int _0^1 \hat{B}_{i_l}(\hat{x}_l) [\kappa ]_{\alpha \alpha }^{1/d} \hat{B}_{j_l}(\hat{x}_l) \,\textrm{d}\hat{x}_l \qquad (l \ne \alpha ) \end{aligned}$$

This reveals the Kronecker sum structure of $\textbf{K}$. For $d=3$ we may write

21 $$\begin{aligned} \textbf{K} = \textsf{M}_3 \otimes \textsf{M}_2 \otimes \textsf{K}_1 + \textsf{M}_3 \otimes \textsf{K}_2 \otimes \textsf{M}_1 + \textsf{K}_3 \otimes \textsf{M}_2 \otimes \textsf{M}_1. \end{aligned}$$

With the definition of the generalized Kronecker sum (8) we can also write,

22 $$\begin{aligned} \textbf{K} = \textsf{K}_1 \mathop {\hat{\oplus }}\textsf{K}_2 \mathop {\hat{\oplus }}\textsf{K}_3. \end{aligned}$$

For this class of problems the diagonalization technique introduced in Section 2.3 represents a direct solver. For general problems, where $\boldsymbol{F}$ is not identity and $\kappa$ is not diagonal and constant, we can apply it as a preconditioner to an iterative linear solver. The robustness of this approach is limited [4].

### Rank-1 Preconditioner

Let us lift the restriction on $\boldsymbol{F}$ being an identity map and $\kappa$ being diagonal and constant on $\Omega$. Clearly, the Kronecker sum structure is lost in that case and the robustness of the preconditioning strategy presented above is rather limited. To improve the robustness of the preconditioner, we need to include some of the model data in the Kronecker sum in Equation (22). To preserve the Kronecker sum structure, we discard the off-diagonal entries in $\textbf{C}$ and find a rank-1 separable representation of its diagonal entries. Following the original work on fast diagonalization [4], we choose to operate directly on the data at quadrature points.

Throughout this work we employ tensor-product Gauss–Legendre quadrature rules with $(p+1)^d$ quadrature points per element. These standard quadrature rules are far from an optimal choice for spline discretizations which feature higher regularity at element interfaces. On the same mesh, the dimension of the space of splines of maximum regularity is lower than that of $C^0$ continuous discretizations, thus resulting in a smaller target space of polynomials for quadrature. Consequently, fewer quadrature points are required for exact integration of the system equations [32]. This observation prompted research towards generation of optimal quadrature rules for isogeometric analysis [3, 32–43]. We do not take advantage of these specialized quadrature techniques that can substantially reduce the number of quadrature points. We assume, however, that the employed quadrature rule has a tensor-product structure. We also note that the choice of quadrature technique used in the formation of the stiffness matrix approximation is independent of the forward problem.

With the choice of a tensor-product quadrature rule with tensor-product weights $W_{\textsf{k}} = (\Pi _{l=1}^d w_{k_l})$ and Cartesian product points $\hat{\boldsymbol{x}}_{\textsf{k}} = (\hat{x}_{k_1},\ldots ,\hat{x}_{k_d})$ indexed by multi-indices $\{ \textsf{k} = (k_1,\ldots ,k_d) \,: \, k_l = 1,\ldots ,n_l,\, l = 1,\ldots , d\}$, we define $\mathcal {C}^{\alpha \beta } \in \mathbb {R}^{n_1\times \cdots \times n_d}$, $\alpha ,\beta =1,\ldots ,d$, to be a tensor of the entries of $[\textbf{C}(\hat{\boldsymbol{x}}_{\textsf{k}})]_{\alpha \beta }$ evaluated at all Cartesian product quadrature points. A separable rank-1 approximation to $\mathcal {C}^{\alpha \beta }$ reads

23 $$\begin{aligned} \mathcal {C}^{\alpha \beta } \approx \textbf{c}^{\alpha \beta }_d \otimes \cdots \otimes \textbf{c}^{\alpha \beta }_1 \qquad \left( \textbf{c}^{\alpha \beta }_k \in \mathbb {R}^{n_k}\right) . \end{aligned}$$

The vectors of coefficients $\textbf{c}^{\alpha \beta }_k$, $k=1,\ldots ,d$, can be found using the separation of variables algorithm used in the original work on fast diagonalization [11, 44]. The separation of variables algorithm is stable as long as the entries in the data tensor $\mathcal {C}^{\alpha \beta }$ are positive, which, for the class of problems at hand and $\alpha =\beta$, is the case. This stability constraint arises from the update rules for the coefficient vectors, which include a square root of a real number. This number is guaranteed to be positive only if the data tensor is entrywise positive (see Algorithm 5 in [44]).

An approximation of the stiffness matrix in Equation (16) neglecting the off-diagonal entries in $\textbf{C}$ ($\alpha \ne \beta )$ is given by

24 $$\begin{aligned} {[}\tilde{\textbf{K}}]_{\textsf{i} \textsf{j}} = \sum _{\alpha =1}^{d} \left( \sum _{k_1=1}^{n_1} \cdots \sum _{k_d=1}^{n_d} \frac{\partial \hat{B}_{\textsf{i}}}{\partial \hat{x}_\alpha } \left( w_{k_1}[\textbf{c}^{\alpha \alpha }_{1}]_{k_1}\cdots w_{k_d}[\textbf{c}^{\alpha \alpha }_{d}]_{k_d}\right) \frac{\partial \hat{B}_{\textsf{j}}}{\partial \hat{x}_\alpha } \right) \end{aligned}$$

In this form, Equation (24) resembles Equation (17). For convenience, we define $\tilde{w}_{k_l}^\alpha := (w_{k_l} [\textbf{c}^{\alpha \alpha }_l]_{k_l})^{1/d}$ as well as

25 $$\begin{aligned} {[}\tilde{\textsf{A}}_{l}^{\alpha }]_{i_l j_l} := \sum _{k_l=1}^{n_l} {\left\{ \begin{array}{ll} \displaystyle \frac{\partial \hat{B}_{i_l}(\hat{x}_{k_l}) }{\partial \hat{x}_\alpha } \tilde{w}_{k_l}^{\alpha } \frac{\partial \hat{B}_{j_l}(\hat{x}_{k_l}) }{\partial \hat{x}_\alpha } & (\text {if } \alpha , \beta = l)\\ {\hspace{9.0pt}} \displaystyle \hat{B}_{i_l}(\hat{x}_{k_l}) \tilde{w}_{k_l}^{\alpha } \hat{B}_{j_l}(\hat{x}_{k_l}) & (\text {if } \alpha , \beta \ne l). \end{array}\right. } \end{aligned}$$

The rank-1 approximation of the stiffness matrix for $d=3$ can then be written as a Kronecker sum,

26 $$\begin{aligned} \tilde{\textbf{K}} = \tilde{\textsf{K}}_1 \mathop {\hat{\oplus }}\tilde{\textsf{K}}_2 \mathop {\hat{\oplus }}\tilde{\textsf{K}}_3, \end{aligned}$$

where $\tilde{\textsf{K}}_l:= \tilde{\textsf{A}}_{l}^{\alpha }$ for $l=\alpha$ and $\tilde{\textsf{M}}_l:= \tilde{\textsf{A}}_{l}^{\alpha }$ otherwise. The implementation requires merely a modification of quadrature weights.

### Rank-*m* Inexact Preconditioner

The rank-1 preconditioner has been shown to be robust with respect to various model data [4]. However, its effectiveness depends on how accurate a rank-1 approximation of the model data is. Therefore, we propose a low-rank inexact preconditioning strategy, which takes into account all of the model data, in particular all components of $\mathcal {C}^{\alpha \beta }$, and permits rank-*m* approximations. Naturally, the increased effectiveness leads to higher computational cost. By leveraging the properties of Kronecker product matrices, in particular fast matrix-vector products, our approach balances cost and effectiveness and thus allows to optimize the performance by choosing a suitable rank *m*.

Instead of a rank-1 approximation of $\mathcal {C}^{\alpha \beta }$ we ask for a *m*-term expansion of this tensor in terms of rank-1 tensors,

27 $$\begin{aligned} \mathcal {C}^{\alpha \beta } \approx \sum _{r=1}^m \textbf{c}^{\alpha \beta }_{d,r} \otimes \cdots \otimes \textbf{c}^{\alpha \beta }_{1,r} \qquad \left( \textbf{c}^{\alpha \beta }_{k,r} \in \mathbb {R}^{n_k}, r=1,\ldots ,m \right) . \end{aligned}$$

The approximation of the stiffness matrix in Equation (16) can be written as a sum of $(d^2m)$ Kronecker products,

28 $$\begin{aligned} {[}\tilde{\textbf{K}}]_{\textsf{i} \textsf{j}} = \sum _{r=1}^m \sum _{\alpha =1}^{d} \sum _{\beta =1}^d \left( \sum _{k_1=1}^{n_1} \cdots \sum _{k_d=1}^{n_d} \frac{\partial \hat{B}_{\textsf{i}}}{\partial \hat{x}_\alpha } \left( w_{k_1}[\textbf{c}^{\alpha \beta }_{1,r}]_{k_1}\cdots w_{k_d}[\textbf{c}^{\alpha \beta }_{d,r}]_{k_d}\right) \frac{\partial \hat{B}_{\textsf{j}}}{\partial \hat{x}_\beta } \right) . \end{aligned}$$

For convenience, we define $\tilde{w}_{k_l,r}^{\alpha \beta }:= (w_{k_l} [\textbf{c}^{\alpha \beta }_{l,r}]_{k_l})^{1/d}$ and the following univariate matrices

29 $$\begin{aligned} {[}\tilde{\textsf{A}}_{l,r}^{\alpha \beta }]_{i_l j_l} := \sum _{k_l=1}^{n_l} {\left\{ \begin{array}{ll} \displaystyle \frac{\partial \hat{B}_{i_l}(\hat{x}_{k_l}) }{\partial \hat{x}_\alpha } \tilde{w}_{k_l,r}^{\alpha \beta } \frac{\partial \hat{B}_{j_l}(\hat{x}_{k_l}) }{\partial \hat{x}_\beta } & (\text {if } \alpha , \beta = l) \\ {\hspace{5.0pt}} \displaystyle \hat{B}_{i_l}(\hat{x}_{k_l}) \tilde{w}_{k_l,r}^{\alpha \beta } \hat{B}_{j_l}(\hat{x}_{k_l}) & (\text {if } \alpha , \beta \ne l)\\ {\hspace{9.0pt}} \displaystyle \frac{\partial \hat{B}_{i_l}(\hat{x}_{k_l}) }{\partial \hat{x}_\alpha } \tilde{w}_{k_l,r}^{\alpha \beta } \hat{B}_{j_l}(\hat{x}_{k_l}) & (\text {if } \alpha =l, \beta \ne l)\\ {\hspace{9.0pt}} \displaystyle \hat{B}_{i_l}(\hat{x}_{k_l}) \tilde{w}_{k_l,r}^{\alpha \beta } \frac{\partial \hat{B}_{j_l}(\hat{x}_{k_l}) }{\partial \hat{x}_\beta } & (\text {if } \alpha \ne l, \beta = l) \end{array}\right. } \end{aligned}$$

The approximation in terms of the univariate matrices $\tilde{\textsf{A}}_{d,r}^{\alpha \beta }$ is then given as

30 $$\begin{aligned} \tilde{\textbf{K}} = \sum _{r=1}^m \sum _{\alpha =1}^{d} \sum _{\beta =1}^d \tilde{\textsf{A}}_{d,r}^{\alpha \beta }\otimes \cdots \otimes \tilde{\textsf{A}}_{1,r}^{\alpha \beta }. \end{aligned}$$

Similarly to previous sections, we define $\tilde{\textsf{K}}_{l,r}:= \tilde{\textsf{A}}_{l,r}^{\alpha \beta }$ $(\alpha ,\beta =l)$ and $\tilde{\textsf{M}}_{l,r}:= \tilde{\textsf{A}}_{l,r}^{\alpha \beta }$ $(\alpha ,\beta \ne l)$. Additionally, we find a univariate matrix of convective type $\tilde{\textsf{D}}_{l,r}:= \tilde{\textsf{A}}_{l,r}^{\alpha \beta }$ $(\alpha =l,\beta \ne l)$. The case $(\alpha \ne l,\beta = l)$ is simply the transpose of $\tilde{\textsf{D}}_{l,r}$. A $(m=1)$-term approximation of the stiffness matrix in Equation (16) for $d=3$ has the form

31 $$\begin{aligned} \begin{aligned} \tilde{\textbf{K}} =&\;\tilde{\textsf{M}}_3 \otimes \tilde{\textsf{M}}_2 \otimes \tilde{\textsf{K}}_1 + \tilde{\textsf{M}}_3 \otimes \tilde{\textsf{K}}_2 \otimes \tilde{\textsf{M}}_1 + \tilde{\textsf{K}}_3 \otimes \tilde{\textsf{M}}_2 \otimes \tilde{\textsf{M}}_1 + \ldots \\&\;\tilde{\textsf{M}}_3 \otimes \tilde{\textsf{D}}_2^\mathrm T\otimes \tilde{\textsf{D}}_1 + \tilde{\textsf{D}}_3 \otimes \tilde{\textsf{M}}_2 \otimes \tilde{\textsf{D}}_1^\mathrm T+ \tilde{\textsf{D}}_3^\mathrm T\otimes \tilde{\textsf{D}}_2 \otimes \tilde{\textsf{M}}_1 + \ldots \\&\;\tilde{\textsf{M}}_3 \otimes \tilde{\textsf{D}}_2 \otimes \tilde{\textsf{D}}_1^\mathrm T+ \tilde{\textsf{D}}_3^\mathrm T\otimes \tilde{\textsf{M}}_2 \otimes \tilde{\textsf{D}}_1 + \tilde{\textsf{D}}_3 \otimes \tilde{\textsf{D}}_2^\mathrm T\otimes \tilde{\textsf{M}}_1, \end{aligned} \end{aligned}$$

where we have dropped the mode index *r* for readability.

The apparent structure of the approximation is convenient as it enables automation of the construction of such operators. We merely need to implement a kernel describing the discrete operator and pass it appropriate data. The formation of the univariate matrices and Kronecker products thereof is executed automatically. The resulting set of Kronecker products is aggregated in a matrix-free linear operator, which implements a matrix-vector product over the sum of the Kronecker products. In this work, we do not further optimize the matrix-vector product, such that the cost of applying the matrix-free linear operator to a vector scales with $\mathcal O({mN^{4/3}})$, where *N* is the size of $\tilde{\textbf{K}}$ and *m* is the number of terms in the data approximation.

It remains to compute the rank-1 tensors necessary for the approximation in Equation (27). As the approach used in fast diagonalization [4] is not suitable in our setting, we choose to use the canonical polyadic decomposition instead. We use the alternating least squares variant of the decomposition [45, 46]. In our experiments, the canonical polyadic decomposition proves to be efficient and robust.

#### Remark 1

A rank-*m* preconditioner may as well be obtained as a low-rank Kronecker approximation of Galerkin matrices, where the model data $\textbf{C}(\boldsymbol{x})$ is projected onto a spline space and the low-rank tensor decomposition is performed on the spline coefficients [16]. This approach has the advantage that the cost of the decomposition can be controlled by the choice of the projection space.

## Inexact Preconditioned Linear Solvers

As the sum of Kronecker products in Equation (28) does not have the Kronecker sum structure present in the direct solver and the rank-1 preconditioner, its inverse cannot readily be applied to a vector of coefficients. This precludes the application of Equation (28) in the standard way as a preconditioner to iterative linear solvers.

To use the rank-*m* approximation in Equation (28) as a preconditioner, we approximate the action of its inverse by an inner iterative solve. Motivated by the fast matrix-vector product, we perform a preconditioned conjugate gradient (PCG) solve up to a predefined tolerance and use the rank-1 preconditioner from Equation (24) to reduce the number of inner iterations in the preconditioning step.

To ensure convergence of the outer linear solver for the forward problem, we turn to a class of iterative linear solvers, which allow for the preconditioner to change from one iteration to another. These linear solvers are often referred to as flexible or inexact preconditioned iterative linear solvers and are mostly variants of the standard iterative linear solvers with minor modifications. The most notable examples are the inexact preconditioned conjugate gradient (IPCG) method [20–22], the Derber-Rosati method [23], the flexible generalized minimal residual method [24] and the nested generalized minimal residual method [25].

For the test problems in this work, the IPCG method will suffice. The solution algorithm is listed in Algorithm 1. It differs from the standard PCG method merely by line 8. In comparison to standard PCG, in order to compute the step size $\beta _n$, we need to store the residual vector from the previous iteration, $\textbf{r}_{n-1}$, and compute the difference to the residual vector in the current iteration, $\textbf{r}_{n} - \textbf{r}_{n-1}$.

The convergence criterion for the inner PCG solve in the *n*-th iteration becomes

32 $$\begin{aligned} {\Vert \textbf{e}_n \Vert }_2 \le \hat{\eta } {\Vert \textbf{r}_n \Vert }_2, \end{aligned}$$

where $\textbf{e}_n$ is the residual vector in the inner iteration and $\textbf{r}_n$ the residual vector in the outer iteration. The parameter $\hat{\eta }$ is dictated by an input parameter $\eta \in [0,1)$ and the condition number of the preconditioner $\textbf{P}$,

33 $$\begin{aligned} \hat{\eta } = \dfrac{\eta }{\sqrt{\textrm{cond}(\textbf{P})}}. \end{aligned}$$

The computation of $\textrm{cond}(\textbf{P})$ for the operator in Equation (28) is inexpensive. The parameter $\eta$ is used to balance the cost of the inner and outer iterations. Setting $\eta$ to a small constant increases the accuracy of the inner solve and in turn – given there is a benefit to increased accuracy of the inner solve – reduces the number of outer iterations. A larger $\eta$ allows for more flexibility of the preconditioner at the cost of an increased number of outer iterations. In the original work, the authors suggest $\eta =0.5$ as the optimal value balancing inner and outer iteration cost. In our setting, we can choose it orders of magnitude smaller, as the matrix-free forward problem is significantly more expensive than the application of the preconditioner. In practice, we can find an optimal $\eta$ on a small test mesh and carry it over to a larger computation, where the cost of the forward problem increases anyways. Similarly, the rank *m* of the data approximation is also carried over to larger computations.

It must be noted that the availability of inexact preconditioned iterative solvers in linear algebra libraries is rather limited. To keep benchmarks convincing, we use our own implementations of PCG and IPCG. The performance of our BLAS implementations is on a par with iterative linear solvers available in Julia.

**Algorithm 1:**
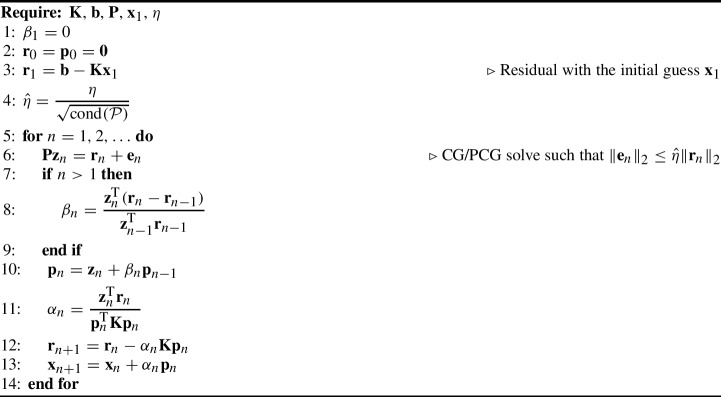
Inexact preconditioned Conjugate Gradient method (IPCG).

## Numerical Examples

In the following, we test the proposed preconditioning strategy for Poisson and linear elasticity problems in two and three dimensions. We emphasize that at no point the system matrices are explicitly assembled. The action of the system matrices on a vector of coefficients is computed using sum-factorization techniques. As shown in Equation (16), the model data includes the geometric mapping and the material properties. We investigate the effect of different geometric mappings, anisotropy and near incompressibility.

We benchmark the setup time of the fast diagonalization preconditioner, the formation time of the stiffness matrix approximation, the total solution time, and the number of iterations of the proposed inexact preconditioned conjugate gradient (IPCG) method against the standard preconditioned conjugate gradient (PCG) method using the fast diagonalization preconditioner or – in the case of linear elastic problems – a block-diagonal fast diagonalization preconditioner.

The models and the open-source Julia implementation are available in our respository at https://taiga.mika.sh/.

### Inexact Preconditioned Solution of the Poisson Problem

**Fig. 1:**
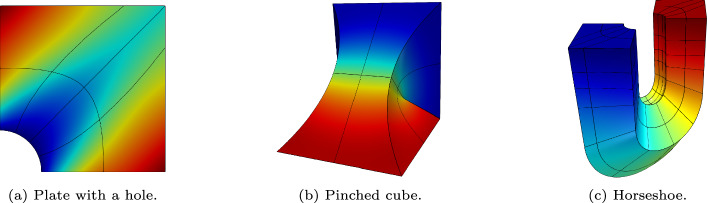
NURBS geometries used in the Poisson benchmarks with meshes and the primary field resulting from one uniform *h*-refinement of the parametric space

Figure 1 illustrates the domains for the Poisson model problem as well as the corresponding meshes and solutions after one uniform *h*-refinement of the parametric space. In the two-dimensional benchmark case the geometric mapping describes the common square plate with a hole shown in Fig. 1a. In the first of the two three-dimensional benchmark cases, the domain is a pinched cube shown in Fig. 1b. This geometry is obtained from the column geometry in Fig. 4 by rotating the top surface by almost ninety degrees around an axis through the column’s center of mass. We emphasize that it does not exhibit any singularities. The second three-dimensional benchmark involves the horseshoe geometry shown in Fig. 1c, which was first presented in [47] and serves as a test case involving a more intricate geometric mapping. We benchmark the Poisson problem on these domains for iso- and anisotropic conductivity tensors, as the condition number of the system matrix increases with increasing ratio of anisotropy.

#### Anisotropic Poisson Equation on the Plate with a Hole Domain

We first consider the anisotropic Poisson equation on the plate with a hole domain with homogeneous boundary conditions. The diagonal coefficients of the conductivity tensor are equal to 1.0, while the off-diagonal coefficients are equal to 0.9. The source term is constant and equal to one. We compute the solution with an absolute tolerance of $\varepsilon _{\textrm{tol}} = 10^{-7}$. The data approximation used to build the preconditioner is computed by the canonical polyadic decomposition with ($m=10$)-terms. The parameter $\eta$ in the IPCG method is $\eta = 10^{-4}$. The solution times in seconds for different levels of *p*- and uniform *h*-refinement are summarized in Table 1. As expected, the solution time increases with an increasing number of elements as well as an increasing polynomial order. While computing times for the IPCG method range between hundreds of milliseconds and tens of seconds, the standard PCG method using fast diagonalization requires seconds to thousands of seconds.

**Table 1:** Total solution times $T_{\textrm{IPCG}}$ and $T_{\textrm{PCG}}$ in seconds for the anisotropic Poisson benchmark problem on the plate with a hole domain solved using the IPCG and the PCG method. The speedup factor *S* is defined as the ratio $T_{\textrm{PCG}} / T_{\textrm{IPCG}}$

$n_{el}$	$p = 2$			$p = 3$			$p = 4$		
	$T_{\textrm{IPCG}}$	$T_{\textrm{PCG}}$	*S*	$T_{\textrm{IPCG}}$	$T_{\textrm{PCG}}$	*S*	$T_{\textrm{IPCG}}$	$T_{\textrm{PCG}}$	*S*
$70\times 35$	0.11	2.12	19.26	0.19	4.64	24.69	0.53	9.12	17.19
$140\times 70$	0.64	12.82	19.94	3.07	28.66	9.34	3.78	42.69	11.29
$280\times 140$	5.22	71.54	13.72	6.26	136.42	21.81	8.86	248.48	28.03
$560\times 280$	26.63	380.36	14.28	46.3	944.13	20.39	53.65	1603.34	29.89

The corresponding speedup factors show a decrease in solution time by one order of magnitude and, given that the number of iterations for the IPCG method remains constant with refinement, tend to increase with the polynomial order and number of elements. Naturally, this trend is disturbed if the number of iterations for the IPCG method increases under refinement as shown in Table 2 for $p=2,3$. The IPCG method requires between two and three iterations to converge in all cases, while the standard PCG method requires between 97 and 269 iterations. As shown in Table 3, the number of inner iterations in the preconditioning step of the IPCG method follows the trends and is of the same order as the number of iterations in the forward problem solved using PCG.

**Table 2:** Number of iterations $N_{\textrm{IPCG}}$ and $N_{\textrm{PCG}}$ for the anisotropic Poisson benchmark problem on the plate with a hole domain solved using the IPCG and the PCG method

$n_{el}$	$p = 2$		$p = 3$		$p = 4$	
	$N_{\textrm{IPCG}}$	$N_{\textrm{PCG}}$	$N_{\textrm{IPCG}}$	$N_{\textrm{PCG}}$	$N_{\textrm{IPCG}}$	$N_{\textrm{PCG}}$
$70\times 35$	2	97	2	110	3	121
$140\times 70$	2	132	3	150	3	165
$280\times 140$	3	174	3	196	3	215
$560\times 280$	3	218	3	245	3	269

**Table 3:** Number of inner iterations in each outer iteration for the anisotropic Poisson benchmark problem on the plate with a hole domain solved using the IPCG method

$n_{el}$	$p = 2$	$p = 3$	$p = 4$
$70\times 35$	[60, 49]	[69, 56]	[88, 72, 69]
$140\times 70$	[88, 68]	[113, 89, 84]	[124, 95, 93]
$280\times 140$	[120, 92, 91]	[136, 98, 100]	[167, 125, 128]
$560\times 280$	[159, 112, 114]	[198, 147, 150]	[216, 164, 165]

Both the IPCG method and the PCG method with fast diagonalization require some setup work prior to their application in the forward problem. The time required to perform that work is primarily related to the computation of the data approximation in both cases. The data approximation for the setup of the PCG preconditioner is computed using the same algorithms as in [4, 11, 44]. As motivated earlier, in IPCG we compute the canonical polyadic decomposition of the data. Table 4 shows the corresponding setup times of the inner fast diagonalization preconditioner $\textbf{P}^{-1}$ and the rank-*m* stiffness matrix approximation $\tilde{\textbf{K}}$ for the Poisson problem on the plate with a hole domain. The setup times of $\textbf{P}^{-1}$ are lower than those of $\tilde{\textbf{K}}$, yet of the same order of magnitude. The cost of setting up $\tilde{\textbf{K}}$ is to be considered as *additional cost* when comparing the discussed IPCG and PCG methods.

**Table 4:** Setup times of the stiffness matrix approximation and the fast diagonalization preconditioner, $T_{\tilde{\textbf{K}}}$ and $T_{\textbf{P}^{-1}}$ in seconds, respectively, for the anisotropic Poisson benchmark problem on the plate with a hole domain

$n_{el}$	$p = 2$		$p = 3$		$p = 4$	
	$T_{\tilde{\textbf{K}}}$	$T_{\textbf{P}^{-1}}$	$T_{\tilde{\textbf{K}}}$	$T_{\textbf{P}^{-1}}$	$T_{\tilde{\textbf{K}}}$	$T_{\textbf{P}^{-1}}$
$70\times 35$	0.09	0.06	0.14	0.11	0.22	0.19
$140\times 70$	0.27	0.33	0.52	0.46	0.92	0.72
$280\times 140$	1.12	0.96	1.97	1.83	3.09	3.06
$560\times 280$	4.15	3.89	9.72	9.21	15.19	14.38

#### Isotropic Poisson Equation on the Pinched Cube Domain

Next, we consider the isotropic Poisson equation on the pinched cube domain with non-homogeneous boundary conditions at the two closest flat surfaces. The conductivity tensor is the identity tensor. The forcing is zero. We compute the solution with an absolute tolerance of $\varepsilon _{\textrm{tol}} = 10^{-7}$. The data approximation used to build the preconditioner is computed by the canonical polyadic decomposition with ($m=10$)-terms. The parameter $\eta$ in the IPCG method is $\eta = 10^{-8}$.

**Table 5:** Total solution times $T_{\textrm{IPCG}}$ and $T_{\textrm{PCG}}$ in seconds for the isotropic Poisson benchmark problem on the pinched cube domain solved using the IPCG and the PCG method. The speedup factor *S* is defined as the ratio $T_{\textrm{PCG}} / T_{\textrm{IPCG}}$

$n_{el}$	$p = 2$			$p = 3$			$p = 4$		
	$T_{\textrm{IPCG}}$	$T_{\textrm{PCG}}$	*S*	$T_{\textrm{IPCG}}$	$T_{\textrm{PCG}}$	*S*	$T_{\textrm{IPCG}}$	$T_{\textrm{PCG}}$	*S*
$5^3$	0.05	0.19	3.81	0.07	0.48	6.62	0.28	1.17	4.2
$10^3$	0.46	3.09	6.75	0.94	8.23	8.76	1.12	13.49	12.0
$25^3$	7.72	78.55	10.17	9.82	131.97	13.44	43.4	547.33	12.61
$50^3$	52.8	490.8	9.3	118.32	1626.58	13.75	274.56	3259.29	11.87

The solution times in seconds for different levels of *p*- and uniform *h*-refinement are summarized in Table 5. Again, the solution time increases with the number of elements and the polynomial order. For the IPCG method, the solution time lies in the range of tens of milliseconds to hundreds of seconds, while the PCG method requires hundreds of milliseconds to thousands of seconds. The speedup factors tend to increase with increasing problem size up to more than one order of magnitude for the larger test cases. Clearly, computationally more expensive preconditioning techniques work best for larger problems if they retain their effectiveness.

**Table 6:** Number of iterations $N_{\textrm{IPCG}}$ and $N_{\textrm{PCG}}$ for the isotropic Poisson benchmark problem on the pinched cube domain solved using the IPCG and the PCG method

$n_{el}$	$p = 2$		$p = 3$		$p = 4$	
	$N_{\textrm{IPCG}}$	$N_{\textrm{PCG}}$	$N_{\textrm{IPCG}}$	$N_{\textrm{PCG}}$	$N_{\textrm{IPCG}}$	$N_{\textrm{PCG}}$
$5^3$	3	38	3	27	3	51
$10^3$	3	50	3	49	3	59
$25^3$	3	102	3	86	4	107
$50^3$	3	113	4	121	5	129

**Table 7:** Number of inner iterations in each outer iteration for the anisotropic Poisson benchmark problem on the pinched cube domain solved using the IPCG method

$n_{el}$	$p = 2$	$p = 3$	$p = 4$
$5^3$	[21, 23, 22]	[13, 15, 14]	[26, 31, 31]
$10^3$	[24, 33, 32]	[22, 28, 27]	[26, 37, 35]
$25^3$	[42, 62, 54]	[33, 54, 50]	[43, 65, 52, 64]
$50^3$	[25, 59, 59]	[12, 43, 46, 44]	[12, 46, 51, 48, 45]

We observe in Table 6 that the IPCG technique requires only three to five iterations to convergence. In comparison, the PCG technique requires between 38 and 129 iterations. Interestingly, the standard PCG technique requires the least iterations in cases with $p=3$. Table 7 shows the number of inner iterations in each outer iteration of the IPCG approach. The average number of inner iterations is roughly half of the number of iterations for the forward problem solved using PCG and fast diagonalization. We note that the parameters for the inexact preconditioner are estimated on the smallest problem and remain unchanged over all benchmarks.

**Table 8:** Setup times of the stiffness matrix approximation and the fast diagonalization preconditioner, $T_{\tilde{\textbf{K}}}$ and $T_{\textbf{P}^{-1}}$ in seconds, respectively, for the isotropic Poisson benchmark problem on the pinched cube domain

$n_{el}$	$p = 2$		$p = 3$		$p = 4$	
	$T_{\tilde{\textbf{K}}}$	$T_{\textbf{P}^{-1}}$	$T_{\tilde{\textbf{K}}}$	$T_{\textbf{P}^{-1}}$	$T_{\tilde{\textbf{K}}}$	$T_{\textbf{P}^{-1}}$
$5^3$	0.25	0.07	0.1	0.2	0.13	0.29
$10^3$	0.27	0.68	0.9	1.94	1.32	2.77
$25^3$	5.11	13.04	10.82	23.92	19.04	48.51
$50^3$	24.18	56.05	71.74	181.6	144.22	361.95

The setup times in Table 8 show, that in three dimensions, the cost of setting up the stiffness matrix approximation is lower than the setup time of fast diagonalization preconditioner, yet still of the same order of magnitude.

#### Anisotropic Poisson Equation on the Horseshoe Domain

Our last benchmark of the Poisson model problem considers the horseshoe domain with mild anisotropy of the conductivity tensor. The diagonal coefficients of the conductivity tensor are equal to 1.0, while the off-diagonal coefficients are equal to 0.25. The forcing is zero and the boundary conditions are non-homogeneous with $u=2$ and $u=3$ on the two flat surfaces of the domain. We compute the solution with an absolute tolerance of $\varepsilon _{\textrm{tol}} = 10^{-7}$. The data approximation used to build the preconditioner is computed by the canonical polyadic decomposition with ($m=13$)-terms. The parameter $\eta$ in the IPCG method is $\eta = 0.05$. The solution times in seconds for different levels of *p*- and uniform *h*-refinement are summarized in Table 9. As expected, the solution time increases with the number of elements and the polynomial order. The speedup factors tend to increase with problem size and exceed one order of magnitude for the largest test cases. The setup times of the operstors are reported in Table 10.

**Table 9:** Total solution times $T_{\textrm{IPCG}}$ and $T_{\textrm{PCG}}$ in seconds for the anisotropic Poisson benchmark problem on the horseshoe domain solved using the IPCG and the PCG method. The speedup factor *S* is defined as the ratio $T_{\textrm{PCG}} / T_{\textrm{IPCG}}$

$n_{el}$	$p = 3$			$p = 4$			$p = 5$		
	$T_{\textrm{IPCG}}$	$T_{\textrm{PCG}}$	*S*	$T_{\textrm{IPCG}}$	$T_{\textrm{PCG}}$	*S*	$T_{\textrm{IPCG}}$	$T_{\textrm{PCG}}$	*S*
$10\times 5\times 30$	1.9	14.1	7.42	10.2	41.93	4.11	20.66	94.17	4.56
$20\times 10\times 60$	31.3	150.08	4.79	82.63	479.16	5.8	139.46	1093.48	7.84
$30\times 15\times 90$	102.36	751.95	7.35	267.88	2354.39	8.79	476.69	4450.67	9.34
$40\times 20\times 120$	567.73	2738.73	4.82	805.45	6499.43	8.07	1297.21	13740.64	10.59

Table 11 shows that the IPCG technique requires between 7 and 9 iterations to convergence. In comparison, the standard PCG technique requires between 109 and 336 iterations. Table 12 shows the number of inner iterations in each outer iteration of the IPCG approach. The average number of inner iterations is less than half of the number of iterations for the forward problem solved using PCG and fast diagonalization. Again, the parameters for the inexact preconditioner are estimated on the smallest problem and remain unchanged throughout the different meshes.

**Table 10:** Setup times of the stiffness matrix approximation and the fast diagonalization preconditioner, $T_{\tilde{\textbf{K}}}$ and $T_{\textbf{P}^{-1}}$ in seconds, respectively, for the anisotropic Poisson benchmark problem on the horseshoe domain

$n_{el}$	$p = 3$		$p = 4$		$p = 5$	
	$T_{\tilde{\textbf{K}}}$	$T_{\textbf{P}^{-1}}$	$T_{\tilde{\textbf{K}}}$	$T_{\textbf{P}^{-1}}$	$T_{\tilde{\textbf{K}}}$	$T_{\textbf{P}^{-1}}$
$10\times 5\times 30$	0.72	1.49	1.86	4.42	4.16	8.3
$20\times 10\times 60$	5.5	12.2	13.86	29.59	23.55	55.41
$30\times 15\times 90$	18.96	42.54	50.6	105.21	73.5	188.02
$40\times 20\times 120$	48.78	133.46	117.08	270.76	203.12	480.82

**Table 11:** Number of iterations $N_{\textrm{IPCG}}$ and $N_{\textrm{PCG}}$ for the anisotropic Poisson benchmark problem on the horseshoe domain solved using the IPCG and the PCG method

$n_{el}$	$p = 3$		$p = 4$		$p = 5$	
	$N_{\textrm{IPCG}}$	$N_{\textrm{PCG}}$	$N_{\textrm{IPCG}}$	$N_{\textrm{PCG}}$	$N_{\textrm{IPCG}}$	$N_{\textrm{PCG}}$
$10\times 5\times 30$	7	109	6	133	8	158
$20\times 10\times 60$	7	163	7	199	8	233
$30\times 15\times 90$	7	205	8	248	9	290
$40\times 20\times 120$	9	237	8	288	8	336

**Table 12:** Number of inner iterations in each outer iteration for the anisotropic Poisson benchmark problem on the horseshoe domain solved using the IPCG method

$n_{el}$	$p = 3$	$p = 4$	$p = 5$
$10\times 5\times 30$	[16, 44, 37, 40	[18, 55, 52,	[18, 67, 69, 59,
	39, 37, 40]	48, 55, 49]	61, 62, 60, 66]
$20\times 10\times 60$	[18, 68, 61, 53,	[18, 84, 84, 72,	[20, 102, 97, 92,
	62, 55, 56]	77, 77, 77]	92, 95, 95, 98]
$30\times 15\times 90$	[20, 87, 81, 76,	[21, 109, 104, 94,	[21, 129, 119, 118, 104,
	74, 75, 74]	97, 101, 91, 101]	111, 116, 113, 112]
$40\times 20\times 120$	[21, 103, 93, 93, 78,	[23, 129, 119, 120,	[23, 153, 140, 124,
	87, 90, 87, 85]	110, 115, 112, 116]	134, 126, 125, 123]

**Fig. 2:**
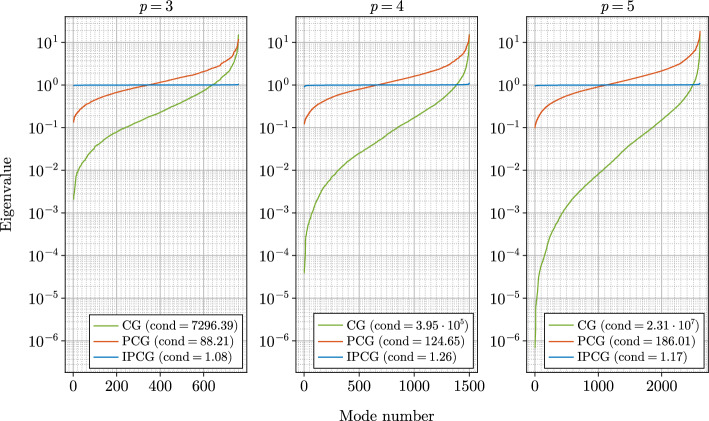
Eigenvalue spectra and condition numbers in the anisotropic Poisson benchmark problem on the horseshoe domain discretized by $4\times 2\times 12$ elements and polynomial degrees $p=3,4,5$. The CG and PCG spectra correspond to no preconditioner and fast diagonalization preconditioner, respectively. The IPCG spectrum assumes that an exact inverse of the stiffness matrix approximation in Equation (30) is applied as preconditioner

**Fig. 3:**
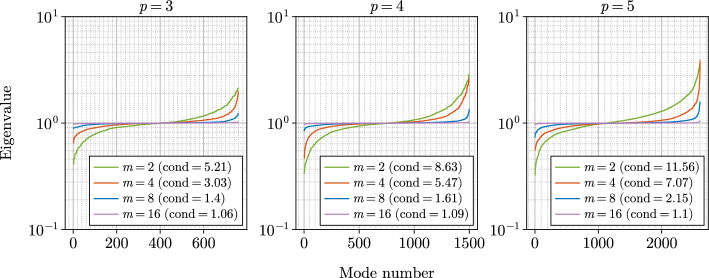
Eigenvalue spectra and condition numbers for IPCG in terms of rank *m* in the anisotropic Poisson benchmark problem on the horseshoe domain discretized by $4\times 2\times 12$ elements and polynomial degrees $p=3,4,5$. The exact inverse of rank *m* stiffness matrix approximation in Equation (30) is applied as preconditioner

In Fig. 2 we show the distribution of the eigenvalues of the stiffness matrix without preconditioning as well as with the fast diagonalization and the inexact preconditioning strategy. The spectrum for the inexact preconditioning strategy is computed assuming an exact inverse of the stiffness matrix approximation in Equation (30). As expected from the low number of outer iterations in the IPCG solver, the eigenvalues cluster around the value of one. Similarly, Fig. 3 depicts the spectrum of the stiffness matrix preconditioned with the inexact preconditioning strategy as a function of the approximation rank *m* showing better conditioning of the system for increasing approximation rank. Eigenvalues at the lower and the upper part of the spectrum deviate from the value of one. The deviation increases with the polynomial order. We also observe that our inexact preconditioning strategy addresses the upper part of the spectrum better than the fast diagonalization preconditioning strategy. In all three spectra the largest eigenvalue of the system preconditioned by the fast diagonalization preconditioner is an order of magnitude larger than the largest eigenvalue in the inexact preconditioning strategy. We emphasize that in the case of our inexact preconditioning strategy the action of the inverse of the stiffness matrix approximation in Equation (30) is evaluated approximately in each iteration. The level of accuracy depends on the residual from the outer iteration. A theoretical analysis of the spectra in each iteration is rather involved and is defferred to future work.

As the benchmarks and trends above show, the results are in favor of the proposed method. If the anisotropy is well pronounced, the geometric mapping is more intricate or the problem size increases, our method outperforms PCG using fast diagonalization significantly. Extreme anisotropy of the thermal conductivity can be observed for example in boron nitride, graphite and graphene materials, where the ratio of the in-plane and the out-of-plane conductivity can reach three orders of magnitude [48].

### Inexact Preconditioned Solution of Linear Elastic Problems

**Fig. 4:**
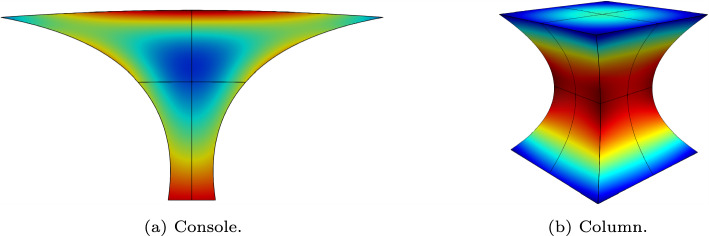
NURBS geometries used in the linear elastic test cases with meshes and the von Mises stress obtained from one uniform *h*-refinement of the parametric space

Due to the simple implementation of the proposed solution technique, our idea can be easily applied to more complex model problems. In elasticity problems, we treat each block in the stiffness matrix in an equal manner as in the Poisson problem. The blockwise approximation is then used as our preconditioner. Fast diagonalization is used to precondition the inner iterations. Figure 4 illustrates the domains used in the following tests, the corresponding meshes and the von Mises stress after one uniform refinement of the parametric space.

Uniform *h*-refinement of the console domain in Fig. 4a introduces distorted elements near the sharp corners located at the top edge. These lead to increasing condition numbers of the system matrix. The benchmarks on the column domain in Fig. 4b focus on the impact of the material parameters on the performance of our method. We consider isotropic, orthotropic and nearly incompressible materials.

#### Plane Stress Linear Elasticity on the Console Domain

We first consider a plane stress problem on the console domain with homogeneous boundary conditions at the narrow bottom edge. At the wide top edge, we apply a traction pointing in the opposite direction of the outward normal with a value of 100. Young’s modulus is $210\times 10^3$ and the Poisson ratio is 0.3. We compute the solution with an absolute tolerance of $\varepsilon _{\textrm{tol}} = 10^{-7}$. The data approximation to build the preconditioner is computed by the canonical polyadic decomposition with ($m=2$)-terms. The parameter $\eta$ in the IPCG method is $\eta = 10^{-4}$. Table 13 summarizes the solution times in seconds for different levels of *p*- and uniform *h*-refinement. As expected, the solution time increases with the number of elements and the polynomial order. For the IPCG method, it lies in the range of tens of milliseconds to tens of seconds, while the standard PCG method with fast diagonalization requires seconds to hundreds of seconds. The speedup factors are in the range of 14 to 49.67 and show a reduction of the solution time by more then one order of magnitude in all cases. Again, as in previous tests, the initial parameters to the IPCG solver remain unchanged throughout all test cases.

**Table 13:** Total solution times $T_{\textrm{IPCG}}$ and $T_{\textrm{PCG}}$ in seconds for the plane stress linear elastic benchmark problem on the console domain solved using the IPCG and the PCG method. The speedup factor *S* is defined as the ratio $T_{\textrm{PCG}} / T_{\textrm{IPCG}}$

$n_{el}$	$p = 2$			$p = 3$			$p = 4$		
	$T_{\textrm{IPCG}}$	$T_{\textrm{PCG}}$	*S*	$T_{\textrm{IPCG}}$	$T_{\textrm{PCG}}$	*S*	$T_{\textrm{IPCG}}$	$T_{\textrm{PCG}}$	*S*
$25^2$	0.08	1.13	14.01	0.19	4.47	23.71	0.26	7.36	28.06
$50^2$	0.44	13.41	30.3	0.82	24.96	30.44	0.84	41.49	49.67
$100^2$	2.62	62.31	23.81	3.36	119.22	35.45	5.02	224.85	44.75
$200^2$	19.93	280.72	14.08	37.19	890.84	23.95	35.81	903.03	25.22

Table 14 shows the number of iterations. The inexact preconditioned technique requires only two iterations to reach convergence in all cases. The standard PCG technique converges in 122 to 698 iterations, while the number of iterations increases significantly with *h*- and *p*-refinement. The effectivity of the IPCG technique is reflected in the speedup factors in each test case. Table 15 shows the number of inner iterations in each outer iteration of the IPCG approach. The average number of inner iterations is more than half of the number of iterations for the forward problem solved using PCG and fast diagonalization.

**Table 14:** Number of iterations $N_{\textrm{IPCG}}$ and $N_{\textrm{PCG}}$ for the plane stress linear elastic benchmark problem on the console domain solved using the IPCG and the PCG method

$n_{el}$	$p = 2$		$p = 3$		$p = 4$	
	$N_{\textrm{IPCG}}$	$N_{\textrm{PCG}}$	$N_{\textrm{IPCG}}$	$N_{\textrm{PCG}}$	$N_{\textrm{IPCG}}$	$N_{\textrm{PCG}}$
$25^2$	2	122	2	150	2	171
$50^2$	2	192	2	239	2	269
$100^2$	2	278	2	340	2	377
$200^2$	2	373	2	698	2	477

**Table 15:** Number of inner iterations in each outer iteration for the plane stress linear elastic benchmark problem on the console domain solved using the IPCG method

$n_{el}$	$p = 2$	$p = 3$	$p = 4$
$25^2$	[98, 92]	[124, 70]	[143, 138]
$50^2$	[157, 47]	[200, 303]	[226, 99]
$100^2$	[232, 116]	[287, 125]	[320, 132]
$200^2$	[308, 192]	[549, 385]	[403, 300]

Again, the setup times given in Table 16 show that in two dimensions, the fast diagonalization preconditioner requires less computational effort than the formation of the approximation of the system matrix. However, we observe that the margin decreases with increasing problem size.

**Table 16:** Setup times of the stiffness matrix approximation and the fast diagonalization preconditioner, $T_{\tilde{\textbf{K}}}$ and $T_{\textbf{P}^{-1}}$ in seconds, respectively, for the plane stress linear elastic benchmark problem on the console domain

$n_{el}$	$p = 2$		$p = 3$		$p = 4$	
	$T_{\tilde{\textbf{K}}}$	$T_{\textbf{P}^{-1}}$	$T_{\tilde{\textbf{K}}}$	$T_{\textbf{P}^{-1}}$	$T_{\tilde{\textbf{K}}}$	$T_{\textbf{P}^{-1}}$
$25^2$	0.08	0.06	0.21	0.17	0.3	0.27
$50^2$	0.29	0.25	0.52	1.09	0.81	0.77
$100^2$	0.95	0.71	2.39	1.72	3.23	3.76
$200^2$	3.14	3.05	5.62	5.54	8.88	8.32

#### Isotropic Linear Elasticity on the Column Domain

All three-dimensional linear elastic benchmarks are performed on the same column domain shown in Fig. 4. In these tests, we focus on different material parameters. First, we consider a simple isotropic material with Young’s modulus of $210\times 10^3$ and the Poisson ratio equal to 0.3. At the bottom surface of the column, we apply homogeneous boundary conditions. At the top surface, we prescribe a constant displacement in the outward normal direction with a value of 0.01. The traction vector is zero on the boundary. We compute the solution with an absolute tolerance of $\varepsilon _{\textrm{tol}} = 10^{-7}$. The data approximation to build the preconditioner is computed by the canonical polyadic decomposition with ($m=2$)-terms. The parameter $\eta$ in the IPCG method is $10^{-8}$. The solution times in seconds for different levels of *p*- and uniform *h*-refinement are summarized in Table 17. As expected, the solution time increases with the number of elements and the polynomial order. It lies in the range of tens of milliseconds to tens of seconds for the IPCG solver, while the standard PCG solver requires hundreds of milliseconds to thousands of seconds. The speedup factors are in the range of 7.14 to 16.3 and show a reduction of the solution time by more then one order of magnitude for the larger test cases. Considering that the standard PCG technique with fast diagonalization performs well in this benchmark case, the speedup factors achieved with the IPCG technique are satisfactory. In addition, they increase with the number of elements and the polynomial order.

**Table 17:** Total solution times $T_{\textrm{IPCG}}$ and $T_{\textrm{PCG}}$ in seconds for the isotropic linear elastic benchmark problem on the column domain solved using the IPCG and the PCG method. The speedup factor *S* is defined as the ratio $T_{\textrm{PCG}} / T_{\textrm{IPCG}}$

$n_{el}$	$p = 2$			$p = 3$			$p = 4$		
	$T_{\textrm{IPCG}}$	$T_{\textrm{PCG}}$	*S*	$T_{\textrm{IPCG}}$	$T_{\textrm{PCG}}$	*S*	$T_{\textrm{IPCG}}$	$T_{\textrm{PCG}}$	*S*
$5^3$	0.07	0.48	7.14	0.18	1.34	7.42	0.27	2.72	9.95
$10^3$	0.77	5.24	6.77	1.28	15.6	12.18	2.13	31.23	14.67
$20^3$	5.49	51.87	9.44	9.99	123.33	12.34	17.57	266.85	15.19
$40^3$	35.85	320.73	8.95	60.46	792.93	13.12	99.82	1626.76	16.3

The number of iterations given in Table 18 shows that the IPCG technique requires only two iterations to reach convergence in all cases. The PCG technique converges in 30 to 64 iterations. Table 19 shows the number of inner iterations in each outer iteration of the IPCG approach. The average number of inner iterations is roughly half of the number of iterations for the forward problem solved using PCG and fast diagonalization.

**Table 18:** Number of iterations $N_{\textrm{IPCG}}$ and $N_{\textrm{PCG}}$ for the isotropic linear elastic benchmark problem on the column domain solved using the IPCG and the PCG method

$n_{el}$	$p = 2$		$p = 3$		$p = 4$	
	$N_{\textrm{IPCG}}$	$N_{\textrm{PCG}}$	$N_{\textrm{IPCG}}$	$N_{\textrm{PCG}}$	$N_{\textrm{IPCG}}$	$N_{\textrm{PCG}}$
$5^3$	2	30	2	33	2	36
$10^3$	2	40	2	43	2	45
$20^3$	2	48	2	52	2	54
$40^3$	2	58	2	61	2	64

**Table 19:** Number of inner iterations in each outer iteration for the isotropic linear elastic benchmark problem on the column domain solved using the IPCG method

$n_{el}$	$p = 2$	$p = 3$	$p = 4$
$5^3$	[16, 17]	[18, 19]	[19, 23]
$10^3$	[20, 23]	[21, 25]	[22, 27]
$20^3$	[23, 28]	[25, 30]	[25, 32]
$40^3$	[26, 34]	[26, 37]	[26, 39]

Table 20 shows the setup times for the block diagonal fast diagonalization preconditioner and the stiffness matrix approximation. The setup times for the approximation are lower that those of the fast diagonalization preconditioner, yet both are still in the same order of magnitude.

**Table 20:** Setup times of the stiffness matrix approximation and the fast diagonalization preconditioner, $T_{\tilde{\textbf{K}}}$ and $T_{\textbf{P}^{-1}}$ in seconds, respectively, for the isotropic linear elastic benchmark problem on the column domain

$n_{el}$	$p = 2$		$p = 3$		$p = 4$	
	$T_{\tilde{\textbf{K}}}$	$T_{\textbf{P}^{-1}}$	$T_{\tilde{\textbf{K}}}$	$T_{\textbf{P}^{-1}}$	$T_{\tilde{\textbf{K}}}$	$T_{\textbf{P}^{-1}}$
$5^3$	0.41	0.55	0.98	1.26	2.32	2.14
$10^3$	2.35	4.4	6.58	11.06	11.57	16.42
$20^3$	20.16	30.7	45.88	74.45	85.6	136.68
$40^3$	71.53	120.06	173.01	283.5	353.35	548.24

#### Orthotropic Linear Elasticity on the Column Domain

In the next example, we consider an orthotropic material. The orthotropic material parameters used in this benchmark are summarized in Appendix A. The boundary conditions are identical to those in Section 5.2.2. We compute the solution with an absolute tolerance of $\varepsilon _{\textrm{tol}} = 10^{-7}$. The data approximation to build the preconditioner is computed by the canonical polyadic decomposition with ($m=2$)-terms. The parameter $\eta$ in the IPCG method is $10^{-8}$. The solution times in seconds for different levels of *p*- and uniform *h*-refinement are summarized in Table 21. The solution time increases with the number of elements and the polynomial order. It lies in the range of tens of milliseconds to over a hundred seconds for the IPCG technique, while the standard PCG method requires hundreds of milliseconds to thousands of seconds.

**Table 21:** Total solution times $T_{\textrm{IPCG}}$ and $T_{\textrm{PCG}}$ in seconds for the orthotropic linear elastic benchmark problem on the column domain solved using the IPCG and the PCG method. The speedup factor *S* is defined as the ratio $T_{\textrm{PCG}} / T_{\textrm{IPCG}}$

$n_{el}$	$p = 2$			$p = 3$			$p = 4$		
	$T_{\textrm{IPCG}}$	$T_{\textrm{PCG}}$	*S*	$T_{\textrm{IPCG}}$	$T_{\textrm{PCG}}$	*S*	$T_{\textrm{IPCG}}$	$T_{\textrm{PCG}}$	*S*
$5^3$	0.07	0.72	11.05	0.17	1.77	10.62	0.26	3.83	14.83
$10^3$	0.62	8.19	13.21	1.23	24.2	19.68	1.97	43.44	22.0
$20^3$	5.13	72.69	14.18	9.56	181.12	18.95	26.13	405.63	15.52
$40^3$	49.82	521.75	10.47	78.63	1301.84	16.56	119.24	2740.58	22.98

As shown in Table 22, the IPCG solver requires only two iterations to convergence in all but one case. In comparison, the standard PCG solver requires between 44 and 104 iterations. Table 23 shows the number of inner iterations in each outer iteration of the IPCG approach. The average number of inner iterations is roughly half of the number of iterations for the forward problem solved using PCG and fast diagonalization. We note that the parameters for the inexact preconditioner are estimated on the smallest problem and remain unchanged over all test cases.

**Table 22:** Number of iterations $N_{\textrm{IPCG}}$ and $N_{\textrm{PCG}}$ for the orthotropic linear elastic benchmark problem on the column domain solved using the IPCG and the PCG method

$n_{el}$	$p = 2$		$p = 3$		$p = 4$	
	$N_{\textrm{IPCG}}$	$N_{\textrm{PCG}}$	$N_{\textrm{IPCG}}$	$N_{\textrm{PCG}}$	$N_{\textrm{IPCG}}$	$N_{\textrm{PCG}}$
$5^3$	2	44	2	50	2	57
$10^3$	2	60	2	67	2	71
$20^3$	2	74	2	82	3	88
$40^3$	2	91	2	98	2	104

**Table 23:** Number of inner iterations in each outer iteration for the orthotropic linear elastic benchmark problem on the column domain solved using the IPCG method

$n_{el}$	$p = 2$	$p = 3$	$p = 4$
$5^3$	[24, 25]	[27, 29]	[29, 33]
$10^3$	[31, 34]	[33, 38]	[35, 41]
$20^3$	[36, 42]	[39, 47]	[40, 51, 49]
$40^3$	[44, 54]	[46, 59]	[47, 62]

The speedup factors in Table 21 are all at least one order of magnitude and tend to increase with problem size. The drop in the speedup factor in the $p=4$ and $20^3$ elements case can be attributed to the increase from two to three iterations for the IPCG solution technique. The number of iterations could be reduced by either increasing the number of terms in the data approximation or, more favorably, by reducing the parameter $\eta$ and thus increasing the accuracy of the inner solve.

#### Nearly Incompressible Linear Elasticity on the Column Domain

The setup of this test is identical to Section 5.2.2 with the exception of the Poisson ratio, which is now set to 0.499 and thus approaches the incompressible limit. We compute the solution with an absolute tolerance of $\varepsilon _{\textrm{tol}} = 10^{-7}$. The data approximation to build the preconditioner is computed by the canonical polyadic decomposition with ($m=2$)-terms. The parameter $\eta$ in the IPCG method is $10^{-8}$. The solution times in seconds for different levels of *p*- and uniform *h*-refinement are summarized in Table 24. The solution time increases with the number of elements and the polynomial order. It lies in the range of hundreds of milliseconds to hundreds of seconds for the IPCG solver, while the standard PCG solver requires seconds to tens of thousands of seconds.

**Table 24:** Total solution times $T_{\textrm{IPCG}}$ and $T_{\textrm{PCG}}$ in seconds for the isotropic, nearly incompressible linear elastic benchmark problem on the column domain solved using the IPCG and the PCG method. The speedup factor *S* is defined as the ratio $T_{\textrm{PCG}} / T_{\textrm{IPCG}}$

$n_{el}$	$p = 2$			$p = 3$			$p = 4$		
	$T_{\textrm{IPCG}}$	$T_{\textrm{PCG}}$	*S*	$T_{\textrm{IPCG}}$	$T_{\textrm{PCG}}$	*S*	$T_{\textrm{IPCG}}$	$T_{\textrm{PCG}}$	*S*
$5^3$	0.26	2.53	9.87	0.57	9.45	16.54	0.99	26.03	26.18
$10^3$	2.05	50.22	24.47	3.57	186.21	52.19	5.13	371.36	72.32
$20^3$	36.98	572.84	15.49	44.38	1561.49	35.19	52.92	3442.1	65.04
$40^3$	516.51	5867.3	11.36	644.73	16090.08	24.96	829.51	35194.21	42.43

As shown in Table 25, the IPCG technique requires only two iterations to reach convergence in all cases. In comparison, the standard PCG technique requires 255 to 1, 428 iterations, where the number of iterations increases with the problem size. Table 26 shows the number of inner iterations in each outer iteration of the IPCG approach. The average number of inner iterations is more than a half of the number of iterations for the forward problem solved using PCG and fast diagonalization. With the exception of the smallest problem, the speedup factors given in Table 24 exceed one order of magnitude. This test shows that the proposed IPCG method performs well even for badly conditioned systems and can significantly reduce the solution time when compared to the standard PCG method using fast diagonalization. Interestingly, for polynomial degrees $p = 2,3,4$, the IPCG solution technique performs best on the mesh with 1, 000 elements.

**Table 25:** Number of iterations $N_{\textrm{IPCG}}$ and $N_{\textrm{PCG}}$ for the isotropic, nearly incompressible linear elastic benchmark problem on the column domain solved using the IPCG and the PCG method

$n_{el}$	$p = 2$		$p = 3$		$p = 4$	
	$N_{\textrm{IPCG}}$	$N_{\textrm{PCG}}$	$N_{\textrm{IPCG}}$	$N_{\textrm{PCG}}$	$N_{\textrm{IPCG}}$	$N_{\textrm{PCG}}$
$5^3$	2	255	2	390	2	557
$10^3$	2	558	2	725	2	835
$20^3$	2	816	2	986	2	1129
$40^3$	2	1084	2	1286	2	1428

**Table 26:** Number of inner iterations in each outer iteration for the isotropic, nearly incompressible linear elastic benchmark problem on the column domain solved using the IPCG method

$n_{el}$	$p = 2$	$p = 3$	$p = 4$
$5^3$	[170, 291]	[240, 356]	[334, 496]
$10^3$	[326, 493]	[421, 587]	[465, 697]
$20^3$	[474, 658]	[556, 829]	[631, 934]
$40^3$	[611, 911]	[736, 1106]	[796, 1263]

## Summary and Conclusions

In this paper, we proposed a matrix-free inexact preconditioning strategy for elliptic partial differential equations discretized by the isogeometric Galerkin method on tensor-product spline spaces. We discussed the construction of our inexact preconditioners as sums of Kronecker product matrices based on separable approximations of the model data including geometry and material parameters. Compared to preconditioners using fast diagonalization, our preconditioners are not generalized Kronecker sums and are not easily invertible. To approximate the action of the inverse on a vector, we used the preconditioned conjugate gradient method as an inner solver. To maintain stability, the forward problem was solved using the inexact preconditioned conjugate gradient method. The preconditioning strategy was applied to Poisson and linear elastic test problems. The models and the open-source Julia implementation are available at https://taiga.mika.sh/.

Our benchmarks showed that the proposed preconditioning strategy significantly reduces the number of iterations required for convergence of the inexact preconditioned conjugate gradient (IPCG) method when compared to the preconditioned conjugate gradient (PCG) method using fast diagonalization. Furthermore, the cost of the matrix-vector products in our preconditioner is low. These combined led to an overall reduction of the solution times by more than one order of magnitude in most cases. The construction of the rank-*m* approximation of the stiffness matrix involves an additional setup cost comparable in magnitude to that of setting up the fast diagonalization preconditioner. In the future, this cost may be avoided by reusing data and, if applicable, non-negative canonical polyadic decompositions from the stiffness matrix approximation directly in the construction of the inner fast diagonalization preconditioner. The robustness of our approach was shown in a nearly incompressible linear elastic problem. While the IPCG method maintained its effectiveness and efficiency, the number of iterations and the solution times for the PCG method increased rapidly. In general, our results showed that the IPCG method is competitive to the PCG method using fast diagonalization.

In the future, it would be interesting to extend our preconditioning strategy to parabolic and hyperbolic problems. While the construction of the inexact preconditioner can remain similar, the overall solution framework must be adapted. In particular, the simple inexact preconditioned conjugate gradient method could be replaced by an inexact preconditioned solver applicable to problems, where the system matrix is not positive definite, for example the flexible generalized minimal residual method (FGMRES). Additionally, some work will be required to assure that the inner solver remains efficient.

## Data Availability

All data used in this work is available at https://taiga.mika.sh/.

## Appendix A Orthotropic Material Parameters

The linear elastic material law used in Section 5.2.3 has the form

A1 $$\begin{aligned} \begin{bmatrix} \sigma _{11} \\ \sigma _{22} \\ \sigma _{33} \\ \sigma _{23} \\ \sigma _{13} \\ \sigma _{12} \end{bmatrix} = \begin{bmatrix} \frac{1 - \nu _{23} \nu _{32}}{D} E_1 &  \frac{\nu _{21} + \nu _{23} \nu _{31}}{D} E_1 &  \frac{\nu _{31} + \nu _{32} \nu _{21}}{D} E_1 &  \cdot &  \cdot &  \cdot \\ \frac{\nu _{12} + \nu _{13} \nu _{32}}{D} E_2 &  \frac{1 - \nu _{13} \nu _{31}}{D} E_2 &  \frac{\nu _{32} + \nu _{31} \nu _{12}}{D} E_2 &  \cdot &  \cdot &  \cdot \\ \frac{\nu _{13} + \nu _{12} \nu _{23}}{D} E_3 &  \frac{\nu _{23} + \nu _{21} \nu _{13}}{D} E_3 &  \frac{1 - \nu _{12} \nu _{21}}{D} E_3 &  \cdot &  \cdot &  \cdot \\ \cdot &  \cdot &  \cdot &  G_{23} &  \cdot &  \cdot \\ \cdot &  \cdot &  \cdot &  \cdot &  G_{13} &  \cdot \\ \cdot &  \cdot &  \cdot &  \cdot &  \cdot &  G_{12} \end{bmatrix} \begin{bmatrix} \varepsilon _{11} \\ \varepsilon _{22} \\ \varepsilon _{33} \\ 2\varepsilon _{23} \\ 2\varepsilon _{13} \\ 2\varepsilon _{12} \end{bmatrix} \end{aligned}$$

with $D = 1 - \nu _{12} \nu _{21} - \nu _{13} \nu _{31} - \nu _{23} \nu _{32} - 2 \nu _{12} \nu _{23} \nu _{31}$. The orthotropic material properties are listed in Table 27.

**Table 27:** Orthotropic material properties used in the linear elastic benchmark case in Section 5.2.3

Parameter	Value
$E_1$	$13.810 \times 10^9$
$E_2$	$0.678 \times 10^9$
$E_3$	$1.311 \times 10^9$
$G_{23}$	$0.255 \times 10^9$
$G_{13}$	$1.012 \times 10^9$
$G_{12}$	$0.753 \times 10^9$
$\nu _{23}$	0.42
$\nu _{13}$	0.46
$\nu _{12}$	0.5
$\nu _{32}$	$\frac{\nu _{23}}{E_2} E_3$
$\nu _{31}$	$\frac{\nu _{13}}{E_1} E_3$
$\nu _{21}$	$\frac{\nu _{12}}{E_1} E_2$

## Footnotes

1 The proposed method is not limited to spline based discretizations. Any tensor-product space is applicable in this context.

## Code Availability

All code used in this work is availabe at https://taiga.milk.sh/.
